# *ANODE*: anomalous and heavy-atom density calculation

**DOI:** 10.1107/S0021889811041768

**Published:** 2011-11-12

**Authors:** Andrea Thorn, George M. Sheldrick

**Affiliations:** a Department of Structural Chemistry, University of Göttingen, Tammannstrasse 4, D-37077 Göttingen, Germany

**Keywords:** anomalous density, heavy-atom density, experimental phasing, computer programs

## Abstract

The new program *ANODE* estimates anomalous or heavy-atom density by reversing the usual procedure for experimental phase determination by methods such as single- and multiple-wavelength anomalous diffraction and single isomorphous replacement anomalous scattering. Instead of adding a phase shift to the heavy-atom phases to obtain a starting value for the native protein phase, this phase shift is subtracted from the native phase to obtain the heavy-atom substructure phase. The required native phase is calculated from the information in a Protein Data Bank file of the structure. The resulting density enables even very weak anomalous scatterers such as sulfur to be located. Potential applications include the identification of unknown atoms and the validation of molecular replacement solutions.

## Abstract 2

The program *ANODE* determines anomalous (or heavy-atom) densities by reversing the usual procedure for experimental phase determination. Instead of adding a phase shift to the heavy-atom phases to obtain a starting value for the native protein phase, this phase shift is subtracted from the native phase to obtain the heavy-atom substructure phase.

## 1. Introduction

The programs *SHELXC*/*D*/*E* (Sheldrick, 2008 ▶, 2010 ▶) adopt a simplified but effective approach to the experimental phasing of macromolecules. *SHELXC* estimates the marker-atom structure factors |*F*_A_| and phase shifts α from the experimental data. The marker atoms are typically heavy metals, halides, selenium or bromine incorporated specifically for phasing, or naturally present metal or sulfur atoms. The |*F*_A_| values are used by *SHELXD* to find the marker-atom positions by integrated Patterson and direct methods. Approximate native phases ϕ_T_ are then estimated by adding the phase shifts α to the calculated phases ϕ_A_ for the marker-atom substructure: 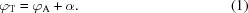
Given high-quality multiple-wavelength anomalous difffraction (MAD) or single isomorphous replacement anomalous scattering (SIRAS) data, the resulting native phases may suffice to give an interpretable map, but for single-wavelength anomalous difffraction (SAD) and SIR phasing these phases will always need to be improved by density modification, *e.g.* with *SHELXE*. In the MAD method, the analysis of data collected at two or more wavelengths close to an absorption edge theoretically enables estimates of |*F*_A_| and α to be obtained, which are limited only by the accuracy of the measured data. In the SAD method, |*F*_A_| is assumed to be proportional to the absolute value of the anomalous difference Δ_ano_ = |*F*_*hkl*_| − |*F*_
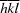
_|, and α is assumed to be 90° when Δ_ano_ is positive and 270° when it is negative. In fact a much better approximation would be Δ_ano_ = |*F*_A_|sinα, with α in the range 0–360°, but it is not possible to deduce two parameters (|*F*_A_| and α) from one observation (Δ_ano_). That SAD phasing works despite these drastic assumptions is a tribute to the power of density modification, though it helps that these approximations hold best for the reflections with the largest anomalous differences.

If the structure and hence ϕ_T_ are known, equation (1) can be rearranged to estimate ϕ_A_: 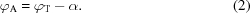
When |*F*_A_| and α originate from SAD data, a map calculated using these values and equation (2) is often colloquially referred to as an ‘anomalous Fourier map’. Such maps were probably first used by Strahs & Kraut (1968 ▶). This approach is, however, equally valid for SIR, MAD and SIRAS phasing, for which ‘heavy-atom density map’ might be a more appropriate description. The program *ANODE* described here simply applies equation (2) to calculate such maps. The phases ϕ_T_ are obtained by a structure factor calculation using the information in a Protein Data Bank (PDB; Berman *et al.*, 2000 ▶) file, and the α and |*F*_A_| values are conveniently provided by the programs *SHELXC* or *XPREP* (from *SHELXTL*; Sheldrick, 2008 ▶), which are currently used to estimate these parameters for experimental phasing. This always creates maps with the same choice of unit-cell origin as the input PDB file, but care may still be needed if the crystal symmetry permits alternative indexing (see §[Sec sec2.4]2.4).

### 1.1. Input and output files

*ANODE* is started by a command line containing a file-name stem and optionally one or more switches:
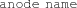
reads a PDB-format file name.ent, or if that is not found name.pdb, and extracts the unit-cell parameters, space-group name and atom coordinates from this file. The file name_fa.hkl from *SHELXC* or *XPREP* is then read to extract the reflection indices and the |*F*_A_| and α values. For the subsequent calculations, it makes no difference whether these originate from SAD, SIR, SIRAS, MAD or radiation-damage-induced phasing experiments. A structure factor calculation using the information from the PDB file generates phases ϕ_T_ for these reflections and ϕ_A_ is then calculated for them using equation (2). The switches control the amount of output required and may be used to truncate the data beyond a specified resolution (−*d*) or multiply the Fourier coefficients by a damping term of the form exp(−8π^2^ *B*sin^2^θ/λ^2^). In practice the default settings for these switches are almost always adequate (Thorn, 2011 ▶). *ANODE* calculates the density map by fast Fourier transform and then derives σ, the square root of the variance of the density, and outputs the following:

(1) The average density (in units of σ) at each type of atom site, *e.g.* ‘S_Met’, using the coordinates from the PDB file.

(2) The heights and coordinates of the unique peaks in the map, and their distances from the nearest atom in the PDB file, taking space-group symmetry and unit-cell translations into account.

(3) A file name.pha containing the map coefficients in a format understood by the program *Coot* (Emsley *et al.*, 2010 ▶) for display of the density.

(4) A file name_fa.res in the same format as written by the program *SHELXD* for SAD or MAD phasing *etc*. This can be input into *SHELXE* (Sheldrick, 2010 ▶) for molecular replacement with SAD phasing (Schuermann & Tanner, 2003 ▶), *e.g.* to reduce the model bias often associated with molecular replacement structure solutions.

(5) A listing file name.lsa.

**Figure 1:**
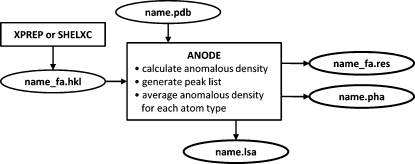
Flow diagram for the *ANODE* program.

Entering anode without a file-name stem on the command line lists the available options. Fig. 1 ▶ shows a flow diagram for *ANODE*.

## 2. Examples

### 2.1. Very weak sulfur-SAD data

**Figure 2:**
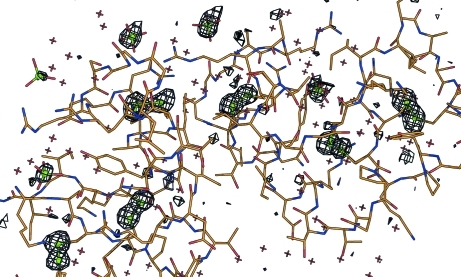
Anomalous density for viscotoxin B2, contoured at 2.8σ. Even though the anomalous signal was too weak for sulfur-SAD phasing, the six disulfide bridges and the S atoms of the sulfate anions are clearly visible in the anomalous density. The density of the sulfate ions is lower because of their higher mobility and because some of them may be partially replaced by phosphate ions.

For attempted phasing based on sulfur as anomalous scatterer, it appears that, even when the Friedel differences are too weak for the structure to be solved by SAD phasing, the S atoms can often be readily identified by *ANODE*. An example is viscotoxin B2 (PDB code 2v9b; Pal *et al.*, 2008 ▶), which resisted all attempts to solve the structure by sulfur-SAD despite the availability of synchrotron data at a wavelength of 1.70 Å and the fact that there are six disulfide bonds in the asymmetric unit (it was in fact solved by *ab initio* direct methods using high-resolution native data from another crystal without using the anomalous data). *ANODE* found a mean density of 9.20σ at the disulfide S atoms and 6.21σ at the S atoms of the six sulfate ions. The next strongest density at an atomic site was 0.84σ. The corresponding anomalous density map is shown in Fig. 2 ▶.

### 2.2. Magic triangle phasing

An attractive alternative to sulfur-SAD and halide soaking (Dauter *et al.*, 2000 ▶) is to soak or co-crystallize with the magic triangle (Beck *et al.*, 2008 ▶), which contains three iodine atoms that make an equilateral triangle with 6.0 Å sides. This structure is easy to recognize and provides much more phasing power than sulfur-SAD. Using the data of Beck *et al.* (2008 ▶) for tetragonal lysozyme (PDB entry 3e3d), *ANODE* obtained average densities of 22.6σ at the 12 iodine sites (four bound I3C molecules), 2.71σ for the SD_Met sites, 2.30σ for the SG_Cys sites and 2.30σ at the S atom of the single HEPES buffer molecule. The next strongest density at an atomic site was 0.54σ. The location of sulfur sites in this way might be useful in tracing a structure phased with the help of I3C.

### 2.3. A MAD example

**Figure 3:**
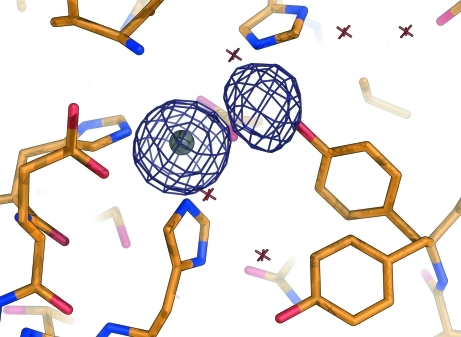
Heavy-atom density from the three-wavelength Zn-MAD data contoured at 3.5σ for thermolysin with excess Zn^2+^, showing the additional partially occupied zinc site about 3.25 Å from the primary zinc site.

At first sight one might expect the density maps created by *ANODE* to show only the atoms with absorption edges straddled by the wavelengths employed in a MAD experiment. However because of their anomalous scattering, other atoms also contribute to the *F*_A_ values, though less strongly than the targeted atoms. The situation becomes clearer when the MAD heavy-atom map is compared with the SAD maps for the different wavelengths used in the MAD experiment. *ANODE* was used to analyse the results of a three-wavelength Zn-MAD experiment on thermolysin (PDB code 3fgd; P. Pfeffer, G. Neudert, L. Englert, T. Ritschel, B. Baum & G. Klebe, in preparation) in which excess Zn^2+^ had been added to ensure full occupancy of the zinc site. All three data sets were collected at beamline 14.2 at the Bessy synchrotron in Berlin to a resolution of 2.06 Å. In addition to strong density at the zinc site, the Ca and S atoms correspond to density maxima. There is also a peak with about 35% of the density of the primary zinc site about 3.25 Å from it. Unlike the calcium and sulfur densities, this density appears as a nearly constant fraction of the primary zinc density, as shown in Table 1 ▶, indicating that it must also be a zinc site (possibly connected to the primary site by one or more O atoms). This confirms the conclusions of Holland *et al.* (1995 ▶) based on the native density and chemical environment of the site. The heavy-atom density map is shown in Fig. 3 ▶.

### 2.4. Inconsistent indexing

When the Laue symmetry is lower than the metric symmetry of the lattice, there are alternative ways of indexing the reflections that are incompatible with each other. Thus the indices in the name_fa.hkl file may not be compatible with those in the data used to generate the PDB file, which may well be from a different crystal. Fortunately, for all but three of the 65 Sohnke space groups, not more than one reorientation matrix needs to be considered. The appropriate matrix is applied to the reflection data when the -i flag is specified on the *ANODE* command line. For the three Sohnke space groups that can each be indexed in four different ways, the flags -i1, -i2 and -i3 may be used. *ANODE* outputs a warning when alternative indexing is possible, so the user does not need to be familiar with such technical details.

## 3. Program availability and distribution

*ANODE* is available as a Fortran source and as precompiled binaries for most modern Windows, Mac and Linux systems as part of the *SHELX* system (http://shelx.uni-ac.gwdg.de/SHELX/), which is distributed free for academic use. The statically linked binaries require no further programs, libraries, data files or environment variables except that the program *SHELXC* (or *XPREP*) is needed to prepare the name_fa.hkl input file for *ANODE*.

## 4. Conclusions

Despite the rather drastic simplifications and approximations involved – in particular the assumption that only one type of anomalous scatterer is present, and the restriction of α to 90 or 270° for SAD data – *ANODE* proves to be a rather effective way to generate and analyse anomalous or heavy-atom densities. *ANODE* appears to work well with appreciably weaker anomalous data than are required for experimental phasing, and should prove useful in the identification of unknown atoms (*e.g.* to distinguish between chloride ions and water molecules), for the validation of molecular replacement solutions (*e.g.* by locating S atoms) or as part of the validation of the final model.

## Figures and Tables

**Table 1:** Heavy-atom densities in σ units from *ANODE* for the three-wavelength Zn-MAD data for thermolysin with excess Zn^2+^ Whereas the ratio of the average density at the calcium sites to that at the zinc site varies with the wavelength for the three SAD experiments because *f*′ for zinc varies strongly, the ratio of the density at the unknown site to that at the zinc site is almost constant (at 35%) for the MAD experiment and for the same data treated as three separate SAD experiments, strongly indicating that this site is also occupied by zinc. Anomalous density is also observed for the methionine S atoms but is barely significant.

Data	Three wavelengths	Inflection point	Peak	High-energy remote
Experiment	MAD	SAD	SAD	SAD
Zn^2+^	82.5	55.7	66.4	56.0
Ca^2+^ (mean)	11.2	15.1	11.1	12.8
SD_Met (mean)	1.8	3.5	2.3	2.9
Unknown	28.5	18.2	24.7	20.1
Ratio Ca^2+^/Zn^2+^	0.136	0.271	0.167	0.229
Ratio unknown/Zn^2+^	0.345	0.326	0.372	0.359
